# Regulation of the *pstSCAB* operon in *Corynebacterium glutamicum* by the regulator of acetate metabolism RamB

**DOI:** 10.1186/s12866-015-0437-1

**Published:** 2015-05-29

**Authors:** Ulrike Sorger-Herrmann, Hironori Taniguchi, Volker F. Wendisch

**Affiliations:** Current adress:, Sandoz, Schaftenau, Austria

**Keywords:** *Corynebacterium glutamicum*, Phosphate starvation, *pstS*, RamB, Phosphorus metabolism, Carbon metabolism, Acetate metabolism, PhoR, GlxR

## Abstract

**Background:**

The *pstSCAB* operon of *Corynebacterium glutamicum*, which encodes an ABC transport system for uptake of phosphate (P_i_), is induced during the P_i_ starvation response. The two-component regulatory system PhoRS is involved in this response, but partial P_i_ starvation induction of *pstSCAB* in a Δ*phoRS* mutant indicated the involvement of additional regulator(s). Regulation of *pstSCAB* also involves the global transcriptional regulator GlxR.

**Results:**

DNA affinity chromatography identified the regulator of acetate metabolism RamB as a protein binding to *pstS* promoter DNA *in vitro*. Gel mobility shift assays and mutational analysis of the *pstS* promoter region revealed that RamB binds to two sites localized at positions −74 to −88 and −9 to +2 with respect to the transcriptional start site of *pstSCAB*. Reporter gene studies supported the *in vivo* relevance of both binding sites for activation of *pstSCAB* by RamB. DNA microarray analysis revealed that expression of many P_i_ starvation genes reached higher levels during the P_i_ starvation response on minimal medium with glucose as sole carbon source than in P_i_ starved acetate-grown *C. glutamicum* cells.

**Conclusions:**

In *C. glutamicum*, RamB is involved in expression control of *pstSCAB* operon. Thus, transcriptional regulation of *pstSCAB* is complex involving activation by the phosphate-responsive two-component regulatory system PhoSR and the regulators of carbon metabolism GlxR and RamB.

## Background

Phosphorus is an essential component of all cells. In bacteria, phosphorus is typically assimilated as inorganic orthophosphate (P_i_) via the reactions of the energy and carbon metabolism, thus, the phosphorus metabolism is closely intertwined with the energy and the central carbon metabolism. An optimal energy and carbon metabolism is possible only with sufficient phosphorus supply. As precursor metabolites for the biosynthesis of amino acids are derived from central carbon metabolism, the interplay of phosphorus and carbon metabolism is of particular interest in amino acid producing *Corynebacterium glutamicum* strains.

P_i_ is taken up into the cell by specific transport systems. When P_i_ becomes scarce, many bacteria induce the synthesis of proteins to use limiting concentrations of P_i_ more efficiently and to make alternative sources of phosphorus accessible. The regulation of the P_i_ starvation response of *Escherichia coli* [1] and *Bacillus subtilis* [2] has been studied in detail. In *E. coli,* the two component regulatory system PhoR-PhoB is responsible for the induction of the P_i_ starvation genes. Under P_i_ starvation conditions, the histidine kinase PhoR phosphorylates the response regulator PhoB and phosphorylated PhoB induces the transcription of at least 38 genes, the so-called PhoB regulon. Among these genes are the *phoBR* operon encoding two component regulatory system, the *pstSCAB-phoU* operon encoding an ABC transporter for high-affinity P_i_ uptake and an regulatory protein, and the *ugpBAECQ* operon encoding an sn-glycerol 3-phosphate ABC uptake system and glycerophosphoryl diester phosphodiesterase. The PhoB regulon in *E. coli* also comprises 21 genes important for uptake and degradation of phosphonates, e.g. the *phnCDEFGHIJKLMNOP* operon. In *B. subtilis*, the P_i_ starvation response is dependent on the two component system PhoP-PhoR for activation of Pho regulon, Spo0A for termination of the P_i_ starvation response and subsequent initiation of sporulation, ResDE for the full induction of the Pho regulon genes and the regulator AbrB. In addition, P_i_ starvation in *B. subtilis* leads to the induction of genes of the general stress response, mediated by σ^B^ and σ^M^ [3–6]. Under P_i_ starvation conditions, *B. subtilis* replaces teichoic acids in the cell-wall with the non-phosphate containing teichuronic acids due to repression of the teichoic acid biosynthesis operons *tagAB* and *tagDEF* and derepression of the teichuronic acid biosynthesis operon *tuaABCDEFGH* [7, 8]*.*

*C. glutamicum* was isolated in 1957 as an L-glutamate excreting bacterium [9] and is used for the large scale biotechnological production of L-glutamate and L-lysine [10, 11]. This bacterium has been engineered for the production of other amino acids such as L-serine [12], L-isoleucine [13], L-valine [14, 15] or L-proline [16]. It has been also successfully engineered to produce derivatives or precursors of amino acids such as 1,4-diaminobutane [17, 18] 1,5-diaminopentane [19], 2-ketoisovalerate [20] and 2-ketoisocaproate [21, 22].

In *C. glutamicum*, phosphorus constitutes 1.5 % to 2.1 % of the cell dry weight [23]. Under P_i_ sufficient conditions, *C. glutamicum* accumulates cytoplasmic and granular polyphosphate [24–26]. Polyphosphate is synthesized by class II polyphosphate kinases [27]. For utilization, it is hydrolysed by exopolyphosphatases [28] and replaces ATP in the reactions of NAD kinase PpnK [29] and glucokinase PpgK [30]. Although intracellular polyphosphate was shown to serve as reservoir of phosphorus [27], expression of a number of genes involved in phosphorus metabolism is induced within 1 h after a shift from P_i_ sufficient to P_i_ limiting conditions [23, 31]. As determined by global gene expression analysis using whole-genome *C. glutamicum* DNA microarrays [31], the P_i_ starvation stimulon comprises among others *pstSCAB* encoding an ABC transporter for high affinity P_i_ uptake, *ugpABCE* encoding an *sn*-glycerol 3-phosphate ABC uptake system, *ushA* encoding a secreted enzyme with UDP sugar hydrolase and 5’nucleotidase activity [32], and the *phoRS* operon encoding for the two component system involved in the P_i_ starvation response of *C. glutamicum* [33]*.* Purified phosphorylated PhoR was shown to bind to the promoters of P_i_ starvation-inducible genes at sites containing a loosely conserved 8-bp direct repeat [34]. Transcriptome analyses of *C. glutamicum* WT and the deletion mutant Δ*phoRS* revealed that the known P_i_ starvation-inducible genes were not induced within 1 h after a shift from P_i_ excess to P_i_ limitation, with the exception of the *pstSCAB* operon, which was still partially induced in the deletion mutant [33]. This indicated that at least one additional regulator besides PhoR is involved in P_i_-dependent regulation of the *pstSCAB* operon in *C. glutamicum*. GlxR, a global cAMP-dependent transcriptional regulator [35–37], was shown to bind to the *pstS* promoter −133 bps to −117 bps upstream of the transcriptional start site and activates the *pstSCAB* operon under phosphate limiting conditions in a carbon source dependent manner [38]. When *glxR* was overexpressed, growth was enhanced under phosphate limiting conditions on glucose as carbon source, but not on acetate [38]. Moreover, a metabolome analysis of *C. glutamicum* grown on acetate or glucose revealed a link between P_i_ limitation and accumulation of glycogen and maltose [39]. However, mutation of GlxR binding site in the *pstS* promoter sequence did not abolish the expression of the reporter gene. This indicated the existence of other factor(s) involved in regulation of *pstS* operon under P_i_ starvation conditions. The aim of this study was to characterize adaptation of *C. glutamicum* to P_i_ starvation in the absence of PhoS-PhoR and to identify additional regulator(s) of *pstSCAB*.

## Results

### Growth of *C. glutamicum* WT and Δ*phoRS* on different phosphorus sources and under P_i_ limiting conditions

To characterize the long-term response of *C. glutamicum* to P_i_ limitation and growth on alternative phosphorus sources, comparative growth experiments were performed with *C. glutamicum* WT and with the deletion mutant Δ*phoRS*, which lacks the two-component regulatory system PhoRS (Table 1) [33]. *C. glutamicum* WT and Δ*phoRS* were pre-cultured for 24 h in CGXII glucose medium without P_i_ in order to exhaust the intercellular phosphorus storages [25, 31] and inoculated into CGXII glucose medium with either a limiting P_i_ concentration of 0.065 mM or with 1 mM of the alternative phosphorus sources of adenosine 5’-monophosphate (5’AMP), L-α-glycerophosphate or UDP-glucose.

**Table 1:** Strains and plasmids used in this study

Strain or plasmid	Relevant characteristic	Reference
*C. glutamicum*		
WT	wild type strain ATCC 13032	[9]
Δ*phoRS*	deletion of the *phoRS* operon encoding the two component system PhoRS	[33]
Δ*ramB*	Deletion of *ramB* encoding regulator of acetate metabolism B	[41]
*E. coli*		
BL21(DE3)	*ompT hsdS*_B_(rB^−^mB^−^) *gal dcm* (DE3)	[64]
DH5α	F^−^ *thi-*1 *endA*1 *hsdr*17(r^−^, m^−^) *supE*44 Δb*lacU*169 (ф80*lac*ZΔM15) *recA*1 *gyrA*96 *relA*1	[65]
Plasmids		
pGEM-T	cloning vector	Promega, WI, USA
pET2	promoter-probe vector	[54]
pET2-RF0	pET2 with *pstSCAB* promoter fragment RF0	This study
pET2-R0F0	pET2 with *pstSCAB* promoterfragment R0F0	This study
pET2-R1F0	pET2 with *pstSCAB* promoter fragment R1F0	This study
pET2-R2F0	pET2 with *pstSCAB* promoter fragment R2F0	This study
pET2-R3F0	pET2 with *pstSCAB* promoter fragment R3F0	This study
pET2-R0F1	pET2 with *pstSCAB* promoter fragment R0F1	This study
pET2-R0F2	pET2 with *pstSCAB* promoter fragment R0F2	This study
pET2-R0F3	pET2 with *pstSCAB* promoter fragment R0F3	This study
pET2-RcFc	pET2 with *pstSCAB* promoter fragment RcFc	This study
pET2-RcFm	pET2 with *pstSCAB* promoter fragment RcFm	This study
pET2-RmFc	pET2 with *pstSCAB* promoter fragment RmFc	This study
pET2-RmFm	pET2 with *pstSCAB* promoter fragment RmFm	This study
pET29*-ramB*-his	Kan^R^; pET29-Histag derivative for over production of RamB with a C-terminal histidine tag	[41]

With 0.065 mM P_i_, which is below the P_i_ concentration of 0.1 mM that supported growth of *C. glutamicum* with a half-maximal growth rate [31], *C. glutamicum* WT showed a doubling time of 0.14 h^−1^ and formed 0.5 g DW l^−1^ biomass whereas the deletion mutant Δ*phoRS* showed a growth defect under P_i_ limiting conditions as expected from previous results (Table 2) [33].

**Table 2:** Growth of *C. glutamicum* WT and Δ*phoRS* on different phosphorus sources

Phosphorus source	Strain	Biomass formed [g/l]	μ [h^−1^]	Duration of lag phase [h]	UDP-glucose hydrolase activity in supernatants [nmol min^−1^ ml^−1^] ^a^
Low Pi, 0.065 mM^b^	WT	2	0.14	0	27
	Δ*phoRS*	1	0.07	6	39
Glycerol-3-phosphate, 1 mM	WT	11	0.16	0	9
	Δ*phoRS*	9	0.11	9	13
5’AMP, 1 mM	WT	9	0.08	11	6
	Δ*phoRS*	7	0.08	34	12
UDP-glucose, 1 mM	WT	9	0.06	39	6
	Δ*phoRS*	8	0.09	63	3

^a^UDP-glucose hydrolase activity was measured after 180 h of cultivation. No UDP-glucose hydrolase activity was detectable (<1 nmol min^−1^ ml^−1^) in supernatants of cells grown under P_i_ sufficient conditions (13 mM)

^b^This concentration is below the P_i_ concentration of 0.1 mM which supports the half-maximal growth rate in *C. glutamicum* [31]

*C. glutamicum* Δ*phoRS* could utilize the alternative phosphorus sources L-α-glycerophosphate, 5’AMP and UDP-glucose, however, it showed longer lag phases, lower growth rates and lower biomass yields than *C. glutamicum* WT (Table 2). As growth of *C. glutamicum* on 5'-AMP and UDP-glucose requires the P_i_ starvation inducible gene *ushA*, which encodes a secreted enzyme with UDP-glucose hydrolase and 5'-nucleotidase activity [32], UDP-glucose hydrolase activity of supernatants of these cultures were measured. While UDP-glucose hydrolase activity could not be detected under P_i_ sufficient conditions (data not shown), supernatants of *C. glutamicum* WT and Δ*phoRS* grown with L-α-glycerophosphate, 5’AMP and UDP-Glucose as sole phosphorus sources showed UDP-glucose hydrolase activity (Table 2). Taken together, PhoRS is not essential for growth with these organophosphates and other regulators apparently allow *C. glutamicum* to induce *ushA* and possibly other genes necessary for the P_i_ starvation response in the absence of PhoRS.

### Deletion analysis of the *pstS* promoter

**Fig. 1:**
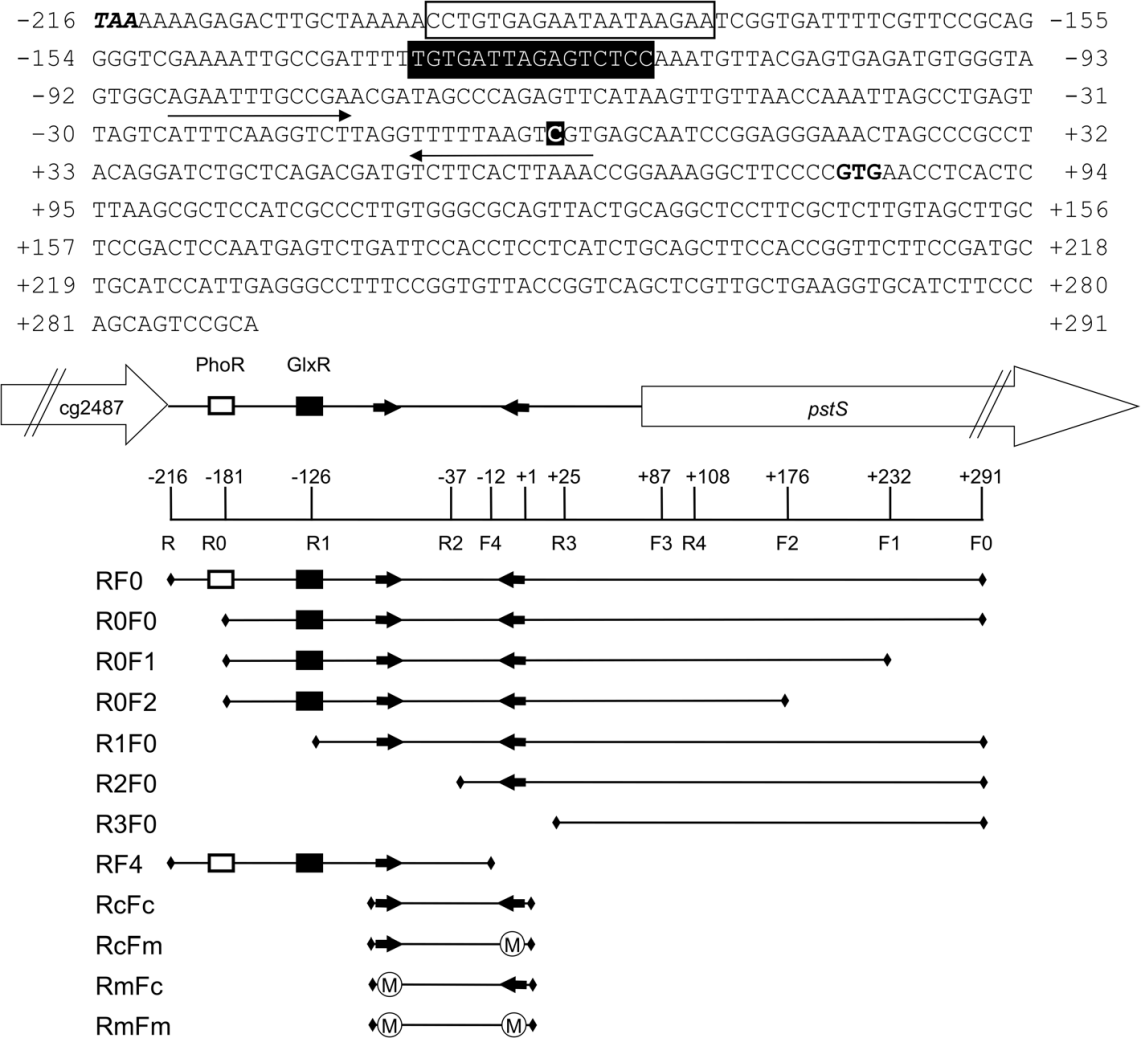
Overview of the *pstS* promoter region and the fragments used in this study. Several DNA fragments were used to analyze RamB binding to the *pstS* promoter in the gel mobility shift assays and the reporter gene assay. The PhoR binding site (open box), GlxR binding site (black box) and two putative RamB binding sites (black arrows) are indicated in the sequence and diagrams. The stop codon of cg2487 (TAA with bold italic), the transcriptional start site of *pstS* (C in a black box), and the *pstS* start codon (GTG in bold) are indicated in the sequence. The number in the diagram indicates the respective position of nucleotide from the transcription start site (+1) of *pstS* and the coverage of each fragment is indicated. A mutation introduced into a RamB binding site is indicated as circled M in the diagram

**Fig. 2:**
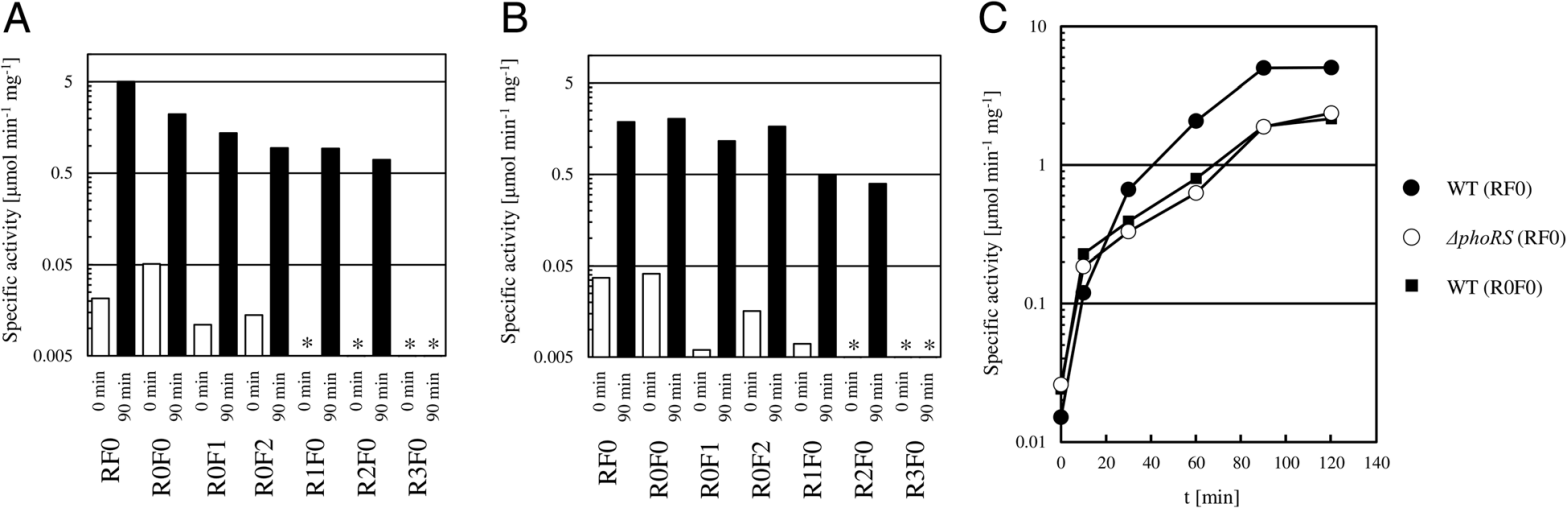
Expression of reporter gene with various promoter fragments in *C. glutamicum* WT and Δ*phoRS*. Expression levels of the fusions in *C. glutamicum* WT (**a**) and in *C. glutamicum* Δ*phoRS* (**b**). Expression levels of the CAT gene fusions were measured before (open bar) and 90 min (filled bar) after the shift from P_i_ sufficient to P_i_ limiting conditions. RF0 to R3F0 indicates the fragment used in the experiment. Expressions are given as specific activity of chloramphenicol acetyltransferase. (*, the specific activity < 0.005) (**c**) Expression levels of the fusions in a time dependent manner. Expression of fusions was measured after a medium shift to medium lacking P_i_. *C. glutamicum* WT (filled) or Δ*phoRS* (open) carrying the promoter fragment RF0 (circle) or R0F0 (square) was used

To identify *cis*-regulatory sequences of the *pstS* promoter for the PhoR-dependent and PhoR-independent control, a deletion analysis of the *pstS* promoter region was performed using different oligonucleotides (Table 3)*.* The *pstS* promoter fragment (RF0) and the promoter fragments either lacking the 5' region (R0F0, R1F0, and R2F0) or the 3' region (R0F1, R0F2, and R0F3) were fused to the promoter-less chloramphenicol acetyl transferase (CAT) gene (Fig. 1). The resulting plasmids pET2-RF0, pET2-R0F0, pET2-R1F0, pET2-R2F0, pET2-R0F1, pET2-R0F2 and pET2-R0F3 were transferred into *C. glutamicum* WT and Δ*phoRS*. Expression of these fusions was assayed before and 90 min after a shift from P_i_ rich to P_i_ lacking medium. The fusion with fragment R3F0 was not expressed as it lacked the previously determined transcriptional start site and the −10 and −35 binding regions of the RNA polymerase (Fig. 2a, b) [33]. All other fusions were expressed and showed P_i_ starvation-inducible expression both in *C. glutamicum* WT and Δ*phoRS* (Fig. 2a, b).

**Table 3:** Oligonucleotides used in this study

Oligonucleotide Sequence (5’ → 3’)	
pstsRforward	CCC*CTCGAG*TAAAAAAGAGACTTGCTAAAAACCT (XhoI)
pstsR0forward	CCC*CTCGAG*TAAGAATCGGTGATTTTCGTTCC (XhoI)
pstsR1forward	CCC*CTCGAG*AGAGTCTCCAAATGTTACGAGTGAA (XhoI)
pstsR2forward	CCC*CTCGAG*CCTGAGTTAGTCATTTCAAGGTCTTA (XhoI)
pstsR3forward	CCC*CTCGAG*GCCCGCCTACAGGATCTGCTCA (XhoI)
pstsF0reverse	CG*TCTAGA*TGCGGACTGCTGGGAAGATG (XbaI)
pstsF1reverse	CG*TCTAGA*CCTCAATGGATGCAGCATCGGAAG (XbaI)
pstsF2reverse	CG*TCTAGA*TCAGACTCATTGGAGTCGGAGCAA (XbaI)
pstsF3reverse	CG*TCTAGA*GTTCACGGGGAAGCCTTTCCGG (XbaI)
pstsF4reverse	CG*TCTAGA*TAAGACCTTGAAATGACTAACTCAGG (XbaI)
pstsFc_reverse	CGGTTTCCCTCCGGATTGCTCACGACTTAAAAACCTA
pstsFm_reverse	CGGTTTCCCTCCGGATTGCGCGCGGAGTAAAAACCTA
pstsRc_forward	CCCGATGTGGGTAGTGGCAGAATTTGCCGAACGAT
pstsRm_forward	CCCGATGTGGGTAGTGGCAGAAGAGGCCGAACGAT
pstsF0biotin	Biotin-TGCGGACTGCTGGGAAGATGCAC

^*^In some cases oligonucleotides were designed to introduce recognition sites for restriction endonucleases (recognition sites in italics)

Expression of the reporter gene fused to the full-length *pstS* promoter in *C. glutamicum* WT (pET2-RF0) was about threefold higher than in *C. glutamicum* Δ*phoRS* (pET2-RF0), while expression of the other fusions did not differ much between WT and Δ*phoRS* (Fig. 2a, b). This indicated that fragment R0F0 lacked a *cis* regulatory sequence required for activation by PhoRS under P_i_ starvation conditions and it is consistent with the finding of a PhoRS binding site in this region [34]. Also the fusions in pET2-R1F0 and pET2-R2F0, which lack the previously determined GlxR binding site, were expressed in *C. glutamicum* WT as well as in Δ*phoRS* upon P_i_ starvation*.*

P_i_ starvation induction of the *pstSCAB* operon is stronger and faster than that of other P_i_ starvation inducible genes of *C. glutamicum* [31] and its induction is partially retained in the absence of PhoRS [33]. Therefore, the time dependent expression from pET2-RF0 and pET2-R0F0 was analyzed in *C. glutamicum* WT and Δ*phoRS* under P_i_ starvation. After a shift from P_i_-sufficient to P_i_-limiting conditions, expression of the *pstS* promoter fusion in pET2-RF0 was induced in *C. glutamicum* WT and Δ*phoRS* before 60 min (Fig. 2c). However, P_i_ starvation induction of the *pstS* promoter in the *phoRS* mutant followed slower kinetics and reached a two to three fold lower level than in *C. glutamicum* WT. On the other hand, induction was very similar between the full-length *pstS* promoter (pET2-RF0) in the *phoRS* mutant and the *pstS* promoter lacking PhoR binding site (pET2-R0F0) in the wild type. Thus, expression control of the *pstS* promoter by PhoRS *in vivo* required the cognate PhoR binding site, which is present in the full-length promoter fragment (RF0), but absent from the 35 nucleotides shorter fragment (R0F0). Furthermore, the fragment R0F0 apparently contains all *cis* regulatory sequences required for P_i_ starvation induction independent of PhoRS. Moreover, the fusions lacking the PhoR and the GlxR binding sites (pET2-R1F0, pET2-R2F0) were still induced under P_i_ starvation conditions. Thus, besides PhoRS, which is required for maximal P_i_ starvation induction of *pstSCAB,* and GlxR, (an) aditional unknown regulator(s) are involved in control of *pstSCAB* expression during adaptation of *C. glutamicum* to P_i_ limitation.

### Identification of RamB as a protein binding to the *pstS* promoter

**Fig. 3:**
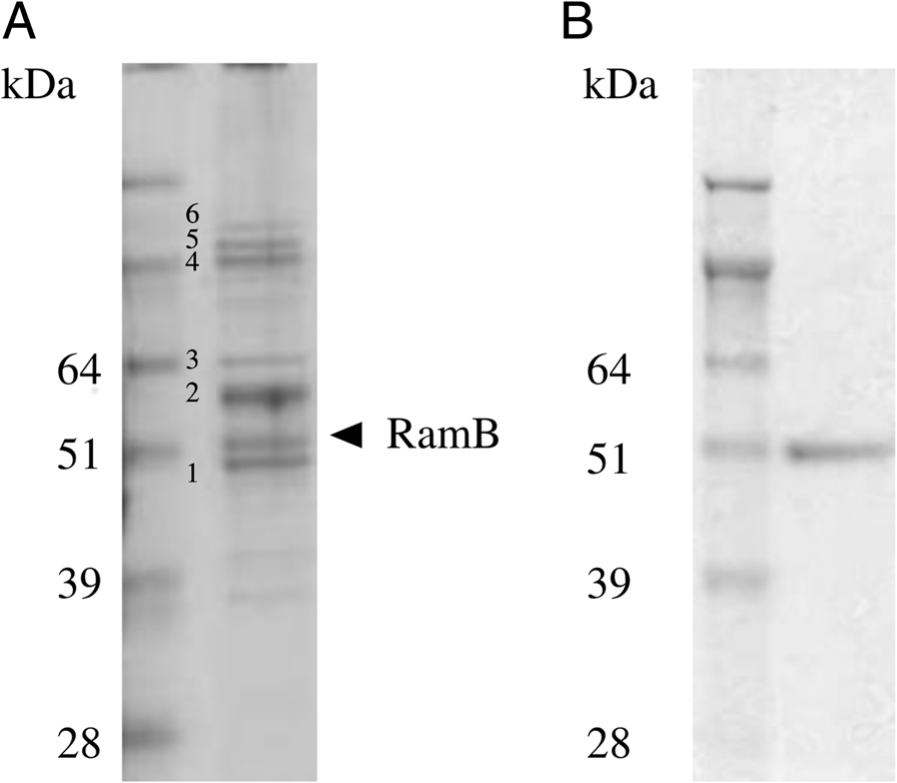
SDS-PAGE images of DNA affinity chromatography experiment and purified RamB protein. (**a**) Proteins eluted from a DNA affinity chromatography experiment using the *pstS* promoter. For the DNA affinity chromatography experiment, the *pstS* promoter fragment R0F0 was used as a probe and incubated with cell extracts of *C. glutamicum* Δ*phoRS* grown under P_i_ sufficient conditions in minimal medium with 4 % (w/v) glucose (Right lane). 1: DNA-polymerase I, 2: Acetyl/propionyl-CoA carboxylase subunit, 3: Acetyl/propionyl-CoA carboxylase subunit, 4: DNA gyrase, 5: DNA-directed RNA polymerase β-subunit, 6: DNA-directed RNA polymerase β’-subunit. Left lane: protein standard Seeblue II prestained Standard (Invitrogen, Karlsruhe) (**b**) Purified His-tagged RamB. His-tagged RamB was over produced in *E. coli*, purified and separated on a 10 % (w/v) SDS-polyacrylamide gel. Gel was stained with Coomassie Blue. Left lane: protein standard Seeblue II prestained Standard (Invitrogen, Karlsruhe), Right lane: purified His-tagged RamB obtained after imidazol elution from a nickel-chelate affinity column

In order to identify (a) regulatory protein(s) binding to the *pstS* promoter region, we coupled the biotinylated *pstS* promoter fragment R0F0 to Dynabeads® streptavidin for DNA affinity purification experiments. DNA affinity chromatography was performed with crude extracts from *C. glutamicum* WT (data not shown) and deletion mutant Δ*phoRS* in CGXII minimal medium with 4 % (w/v) glucose (Fig. 3a). In these experiments, a number of proteins bound to the promoter DNA fragment. By tryptic finger print analysis using MALDI-TOF mass spectrometry, some of these proteins could be identified. Among proteins binding the promoter DNA in a sequence-independent manner (e.g. subunits of RNA polymerase or topoisomerase) the transcriptional regulator RamB was identified (Fig. 3a). The regulator of acetate metabolism RamB is known to repress transcription of the *pta-ack* operon, the *aceA* and *aceB* genes encoding enzymes for acetate activation and of the glyoxylate cycle [40, 41]. Therefore, the DNA affinity chromatography experiments were repeated using crude extracts of *C. glutamicum* WT cultivated on acetate minimal medium under P_i_ starvation conditions. As a result, GlxR and RamB were found to bind to the full-length *pstS* promoter DNA (data not shown). Binding of RamB to the *pstS* promoter DNA suggested its involvement in direct control of the *pstSCAB* operon.

### Purified RamB binds to two binding motifs in the *pstS* promoter *in vitro*

RamB binding sites (AA/GAACTTTGCAAA) are present upstream of many genes encoding enzymes of the central carbon metabolism that belong to the acetate stimulon [41]. However, a RamB binding site within the *pstS* promoter region has not yet been reported. Inspection of the *pstS* promoter DNA suggested the occurrence of two partially conserved RamB binding sites, motif A and motif B: *AGAA*-*TTTGC*CG*A* (−74 to −88) and the reverse complement of *A*CG*ACTT*-AA*AAA* (+2 to −9).

**Fig. 4:**
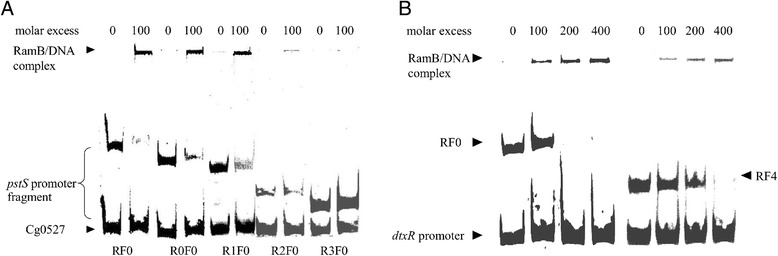
Binding of RamB to various *pstS* promoter fragments. (**a**) Gel shift assay with RamB and the fragment of the *pstS* promoter lacking 5' region. RamB protein (0, 100 fold molar excess) was incubated with the full-length *pstS* promoter (RF0, 507 bp, 15 nM) or the different fragments of the *pstS* promoter lacking 5' region (R0F0, R1F0, R2F0, R3F0, final concentrations 61 nM – 15 nM) and applied for native polyacrylamide gel electrophoresis. A 185 bp promoter fragment of cg0527 served as a negative control. (**b**) Gel shift assay with RamB and the fragment of the *pstS* promoter lacking 3' region. RamB protein (0, 100, 200, 400-fold molar excess) was incubated with the *pstS* promoter (RF0, 507 bp, 15 nM) or fragment of the *pstS* promoter lacking 3' region (RF4, 230 bp, 33 nM) and applied for native polyacrylamide gel electrophoresis. A 122 bp promoter fragment of *dtxR* served as a negative control

In order to test whether RamB directly binds to the *pstS* promoter DNA, band shift assays with purified RamB were performed. RamB containing a C-terminal His-Tag was overproduced in *E. coli* BL21 (DE3) and purified to apparent homogeneity by affinity chromatography (Fig. 3b). Gel shift assays showed that RamB bound with a high affinity to the full-length *pstS* promoter, but not to the negative control fragment cg0527 (Fig. 4a). Gel shift assays with the different fragments of the *pstS* promoter lacking the 5' region (RF0, R0F1, R1F0, R2F0, R3F0) showed binding of RamB to respective DNA fragments except for the fragment R3F0, which lacked both of the predicted RamB binding sites (Fig. 4a). RamB bound weaker to the fragment R2F0, which contains one of the predicted binding site (motif A), than to other fragments which contain both of the predicted binding sites (RF0, R0F0, R1F0). Similarly, the affinity of RamB to fragment RF4, which contains only one of the predicted RamB binding site (motif B), was weaker than that to the full-length *pstS* promoter fragment (RF0) (Fig. 4b). These results suggested the presence of two RamB binding sites in the full-length *pstS* promoter fragment.

**Fig. 5:**
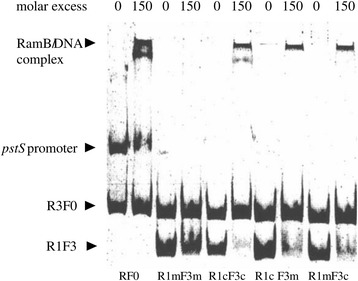
Binding of RamB to various *pstS* promoter fragments. RamB protein (0, 150 fold molar excess) was incubated with the full-length *pstS* promoter fragment (RF0, 5 nM, 507-bp) or the partial length *pstS* promoter fragments (RmFm, RcFm, RmFc, RcFc, 61 nM, 124 bp) and applied for native polyacrylamide gel electrophoresis. A 267 bp fragment of R3F0, which lacked both of RamB binding sites, served as a negative control

A mutational analysis was performed to determine whether both of the partially conserved RamB binding motifs are required for interaction of RamB with the *pstS* promoter. Mutations of RamB binding motif A (AGAA***GAG***GCCGA instead of AGAATTTGCCGA in fragment RmFc), RamB binding motif B (***G***C***G***G***GAG***TAAAAA instead of TCAGACTTAAAAA in fragment RcFm), or of both RamB binding motifs (in fragment RmFm) were introduced into the *pstS* promoter fragment RcFc, which contained both putative binding sites within a 124 bp region (Fig. 1). RamB did not bind to the fragment RmFm containing both mutated binding sites. RamB interacted stronger with non-mutated fragment RcFc than with the fragments RcFm and RmFc, each only containing one intact binding site (Fig. 5). Thus, both binding sites contribute to binding of RamB to the *pstS* promoter *in vitro*.

### Role of RamB sites for regulation of the *pstS* promoter *in vivo*

In order to determine the role of RamB for P_i_ starvation induction of the *pstS* promoter *in vivo*, expression of *pstS* promoter fusion to the promoter-less CAT reporter gene was analyzed in *C. glutamicum* WT on different carbon sources after a shift from P_i_-sufficient to P_i_ starvation conditions. These medium shift experiments were performed with minimal medium containing either 4 % (w/v) glucose or 2 % (w/v) potassium acetate as sole carbon source. Expression of the *pstS* promoter fusion R0F0 after a shift from P_i_-sufficient to P_i_ starvation conditions was higher on glucose than on acetate (2.10 compared to 0.22 μmol min^−1^ mg^−1^, Table 4). P_i_ starvation induced expression of the fusion with the shorter RcFc promoter fragment, which lacks the PhoR and GlxR binding sites, and induction was six fold higher onglucose than on acetate (0.61 as compared to 0.10 μmol min^−1^ mg^−1^, Table 4). When mutations were introduced in only one of RamB binding sites (fragments RmFc and RcFm), expression was reduced both on glucose and acetate. The RmFm fusion carrying mutations in both RamB binding sites showed almost no activity after medium shift on both carbon source (Table 4).

**Table 4:** Expression of various *pstS* promoter fragment *cat* fusions in *C. glutamicum* WT

Promoter fragment in transcriptional fusion	Carbon source	sp. act. of chloramphenicol acetyltransferase [μmol min^−1^ mg^−1^]^a^	
		0 min	90 min^b^
R0F0	Glucose	0.02	2.10
	Acetate	0.01	0.22
RcFc	Glucose	<0.005	0.61
	Acetate	<0.005	0.10
RcFm	Glucose	<0.005	0.12
	Acetate	<0.005	0.01
RmFc	Glucose	<0.005	0.10
	Acetate	<0.005	0.01
RmFm	Glucose	<0.005	0.02
	Acetate	<0.005	<0.005

^a^At least three determinations of two independent cultivations were performed. Average values are given with experimental imprecision < 20 %

^b^The specific activity of chloramphenicol acetyltransferase was measured 0 and 90 min after a shift from P_i_ sufficient to P_i_ limiting conditions

In addition, expression of the *pstS* promoter fusion R0F0 was assayed in the deletion mutant Δ*ramB* growing in 4 % (w/v) glucose as a carbon source before and after P_i_ starvation induction. Before P_i_ starvation, expression of the *pstS* promoter fusion was low, both in WT and in Δ*ramB* (0.02 and 0.02 μmol min^−1^ mg^−1^, respectively), while P_i_ starvation induction was higher in WT as compared to Δ*ramB* (2.10 and 1.23 μmol min^−1^ mg^−1^, respectively, data not shown). Taken together, RamB as well as both RamB binding sites are important for P_i_ starvation induction of the *pstS* promoter in *C. glutamicum in vivo*.

### Comparison of P_i_ starvation inducible gene expression on glucose and acetate minimal medium

As P_i_ starvation induction of the *pstS* promoter differed with respect to the carbon source, DNA microarray analysis was performed to compare the gene expression profile on minimal medium containing either glucose or acetate during the P_i_ starvation response. *C. glutamicum* cells growing exponentially on glucose or acetate minimal medium with 13 mM P_i_ were shifted to minimal medium containing either glucose or acetate but lacking P_i._ RNA was prepared 90 min after the medium shift. As expected for acetate dependent regulation in *C. glutamicum* [41], the DNA microarray analysis revealed two to 100 fold higher mRNA levels for genes belonging to the acetate stimulon on acetate than on glucose: *pta* encoding phosphotransacetylase, *aceA* and *aceB* encoding isocitrate lyase and malate synthase, *pck* encoding gluconeogenetic PEP carboxykinase, *acn* encoding aconiatase and *gltA* encoding citrate synthase (Table 5). Expression of *ramB* was about four fold higher on glucose than on acetate due to autoregulation by RamB and control by RamA [40]. Expression of genes of the *pstSCAB* operon was higher on glucose than on acetate in response to P_i_ starvation, which is consistent with the *pstS* promoter fusion experiments in this study (Table 5). In addition, expression of other genes belonging to the P_i_ starvation stimulon reached higher levels on glucose than on acetate: *ushA* encoding 5’-nucleotidase, *psiB* encoding a putative alkaline phosphatase, *phoH1* encoding a putative ATPase, cg1224 encoding a PhnB-like protein, *pctC* of the *pctABCD* operon encoding an ABC transport system and *ugpA* and *ugpE* of the *ugpEABC* operon encoding an glycerol-3-phosphate uptake system (Table 5). Unlike other genes of the P_i_ starvation stimulon, expression of *phoS* and *phoR* encoding phosphate sensor kinase and its response regulator was lower on glucose than on acetate during P_i_ starvation (Table 5).

**Table 5:** Genes differentially expressed in either glucose or acetate minimal medium cultures of *C. glutamicum* WT after a shift from P_i_-sufficient to P_i_-limiting conditions

Gene identifier	Annotation^a^	Relative mRNA level^b^ glucose/acetate
cg2560	*aceA*, isocitrate lyase	0.01
cg2559	*aceB*, malate synthase	0.05
cg3169	*pck*, phosphoenolpyruvate carboxykinase	0.22
cg3048	*pta*, phosphoacetyltransferase	0.27
cg2887	*phoR*, phosphate response regulator	0.33
cg1737	*acn*, aconitase	0.34
cg0949	*gltA*, citrate synthase	0.46
cg2406	*ctaE*, cytochrome *aa*_3_ oxidase, subunit	0.47
cg2888	*phoS*, phosphate sensor kinase	0.47
cg2843	*pstB*, P_i_ ABC transporter, ATPase	2.0
cg1569	*ugpE*, glycerol 3-phosphate ABC transporter, permease	2.1
cg1224	*phnB1*, PhnB-like protein	2.3
cg0397	*ushA*, UDP sugar hydrolase/5’-nucleotidase	2.4
cg0444	*ramB*, regulator of acetate metabolism B	3.5
cg1647	*psiB*, putative alkaline phosphatase	4.2
cg3393	*phoC*, putative secreted phosphoesterase	4.3
cg0085	*phoH1*, ATPase	5.2
cg1650	*pctC*, ABC transporter, permease	5.2
cg2868	*nucH*, putative nuclease	5.4
cg0812	*accD1*, acetyl-CoA carboxylase subunit	11.3
cg1568	*ugpA*, glycerol 3-phosphate ABC transporter, permease	31.2

^*a*^Gene identifiers and annotations are given according to BX927147

^*b*^The mRNA levels were derived from two independent cultivations

## Discussion

Here we have shown that RamB is involved in expression control of the *pstSCAB* operon during the P_i_ starvation response of *C. glutamicum.* The two component regulatory system PhoR-PhoS is neither essential for P_i_ starvation induction of *pstSCAB* nor for growth on media with the organophosphates glycerol-3-phosphate, 5’-AMP and UDP-glucose as sole phosphorus source. However, PhoR-PhoS ensures rapid and maximal P_i_ starvation induction of *pstSCAB*. The regulator of acetate metabolism RamB was shown to bind to two binding sites in the *pstS* promoter fragment *in vitro* and both of two binding sites were shown to influence the activity of the *pstS* promoter fragment *in vivo* by reporter gene assay. P_i_ starvation induction of the *pstS* promoter fragment reached 10 fold higher levels on glucose minimal medium than on acetate minimal medium. Microarray experiments showed that P_i_ starvation induction of *ramB* and the P_i_ starvation stimulon including *pstSCAB* reached higher RNA levels with glucose as carbon source than with acetate as carbon source. These findings support and extend a regulatory link between phosphorus and carbon metabolism in *C. glutamicum* [38, 39].

The regulator of acetate metabolism RamB represses transcription of the *pta-ack* operon and the *aceA* and *aceB* genes, which encode enzymes for acetate activation and for the glyoxylate cycle [41]. Deletion and mutation analysis of the promoter regions of these genes allowed identifying conserved 13-bp motifs as RamB binding sites [41]. A bioinformatics analysis of the genome sequence revealed that variants of the *cis*-regulatory motif for RamB binding were identified upstream of *aceA, aceB, pta-ack* and also occur in the promoter regions of 28 other genes, 11 of which were differentially expressed in acetate- and glucose-grown *C. glutamicum* cells. These genes code for enzymes of e.g. glucose uptake, glycolysis, glucoeneogenesis, anaplerosis and the tricarboxylic acid cycle [41]. While this bioinformatic analysis searched for variants of the RamB binding site (AA/GAACTTTGCAAA or its complement) with maximal mismatches of two nucleotides [41], the newly identified RamB binding sites in the *pstSCAB* promoter were not recognized previously as they contain 3 (AGAA*-*TTTGC*CG*A) and 5 mismatches (complement of A*CG*ACTT*-AA*AAA)), respectively. Mutational analysis of the RamB binding sites in the *pstS* promoter fragment showed that RamB binds to both of the newly identified RamB binding sites *in vitro* and that both binding sites are relevant for regulation of the *pstS* promoter under P_i_ limiting condition *in vivo*. Thus, RamB appears to activate *pstSCAB* expression under P_i_ limiting conditions. While RamB mostly represses its target genes, RamB was shown to activate *aceE* encoding the E1p subunit of the pyruvate dehydrogenase complex [42].

GlxR also links regulation of carbon and phosphorus metabolism in *C. glutamicum*. GlxR is known to regulate more than 100 genes and is one of the global hubs within the *C. glutamicum* gene-regulatory network [35]. GlxR was shown to bind to the *pstS* promoter in a cAMP-dependent manner *in vitro* [38] and the interaction of GlxR with *pstS* promoter DNA was higher on glucose than on acetate as carbon source in *C. glutamicum* [38, 43]. In this study, expression of the reporter gene fusion with the full length *pstS* promoter (RF0) was higher under P_i_ starvation conditions than expression of the fusion lacking the PhoR binding site (R0F0) and even higher than expression of the fusion lacking both the PhoR and GlxR binding sites (R1F0) (Fig. 2). Thus, the three transcriptional regulators PhoR, GlxR and RamB synergistically activate expression of the *pstS* operon under P_i_ starvation conditions.

GlxR, RamA and RamB also regulate transcription of their genes, e.g. GlxR activates *ramA* and represses *ramB* [35], RamA activates *ramB* [40] and GlxR, RamA and RamB show negative autoregulation [44–46]. Moreover, a number of target genes of RamB and RamA are also regulated by GlxR, e,g, *adhA* and *ald* encoding alcohol dehydrogenase and acetaldehyde dehydrogenase [41] as well as *gltA* encoding citrate synthase [44] are repressed by both GlxR and RamB, but activated by RamA, *rpf2* encoding resuscitation promoting factor 2 is activated by RamA and GlxR, but repressed by RamB [45]. Negative autoregulation of RamB, carbon source-dependent activation of *ramB* by RamA [40] and cAMP-dependent activation of *ramB* by GlxR fine-tune regulation of carbon metabolism and also serve to integrate regulation of carbon and phosphorus metabolism in *C. glutamicum*.

Regulation of *pstSCAB* in *C. glutamicum* is complex, involves at least three transcriptional regulators: PhoR [33], GlxR [38] and RamB (this study) and differs from regulation of the *pstS* promoter in *M. tuberculosis*, *E. coli* and *B. subtilis*. Notably, in the related actinomycete *Mycobacterium tuberculosis* transcription of the *pst* operon is not induced upon P_i_ starvation. Since *M. tuberculosis* can replicate in the phagosomes of macrophages, an acidic and P_i_ poor environment, constitutive expression of *pst* may be a consequence of this intracellular life style [47]. In *E. coli,* the *pstS* promoter is regulated by integration host factor (IHF) and PhoB [48, 49], whereas this promoter is regulated in *B. subtilis* by PhoP [50].

## Conclusions

In *C. glutamicum*, RamB is involved in expression control of the *pstSCAB* operon and two binding sites are relevant for activation by RamB *in vitro*. These finding support the notion that phosphorus and carbon metabolism in *C. glutamicum* are regulated in dependence of each other. Transcriptional regulation of *pstSCAB* is complex involving activation by the phosphate-responsive two-component regulatory system PhoSR and the regulators of carbon metabolism GlxR and RamB.

## Methods

### Bacterial strains, media, and growth conditions

Bacterial strains and plasmids used in this work are listed in Table 1. *E. coli* DH5α (Invitrogen) was used as host during the construction of recombinant plasmids and grown aerobically at 37 °C on a rotary shaker (120 rpm) in Luria-Bertani (LB) medium [51]. *E. coli* BL21 (DE3) was used for overproduction of RamB protein and grown aerobically at 37 °C on a rotary shaker (120 rpm) in LB medium. When appropriate, ampicillin was added at a concentration of 100 μg/ml. *C. glutamicum* wild-type strain ATCC 13032 (WT) and the Δ*phoRS* deletion mutant [33] were grown aerobically at 30 °C on a rotary shaker (120 rpm) in 500 ml baffled shake flasks with 60 ml BHI complex medium or CGXII minimal medium [52]. *C. glutamicum* cells were inoculated from 5 ml LB medium overnight culture to an optical density at 600 nm (OD_600_) of 0.6 in 60 ml CGXII-medium with 0.03 g/l protocatechuic acid as iron chelator and 40 g/l glucose or 20 g/l sodium acetate as carbon and energy source. For medium shift experiments, cells were harvested 14–18 h after inoculation by centrifugation at 4 °C, washed with CGXII without P_i_ and carbon sources, and inoculated in 60 ml CGXII medium with sufficient P_i_ (13 mM) to an optical density at 600 nm (OD_600_) of 0.6. These main cultures were cultivated until OD_600_ of 4 – 5 h. The cells were harvested and either stored at −20 °C for further analysis or washed with CGXII without P_i_ and carbon source, and resuspended in an equal volume of fresh CGXII medium that contained either a limiting P_i_ concentration (0.065 mM) or no P_i_. After incubation at 30 °C for 10, 30, 60, 90 and 120 min in the P_i_ low or P_i_ free medium, cells were harvested and stored at −20 °C for further analysis. For comparative growth experiments on different phosphorus sources, *C. glutamicum* cells growing exponentially on CGXII medium with sufficient P_i_ (13 mM) were inoculated in 60 ml P_i_-free CGXII medium to an OD_600_ of 0.6 and cultured for 24 h at 30 °C to deplete intracellular polyphosphate storage. Afterwards, these cells were harvested, washed with CGXII without P_i_ and carbon source, and inoculated to an OD_600_ of 0.6 in CGXII medium containing either 0.065 mM P_i_, 1 mM adenosine 5’-monophosphate (5’AMP), 1 mM L-α-glycerophosphate or 1 mM UDP-Glucose as sole phosphorus source.

### Preparation of supernatants and assay to determine UDP-glucose hydrolase activity

Cell cultures were centrifuged for 10 min at 5,000 g and 4 °C. Supernatants were passed through a 0.2 μm sterile filter and concentrated about 50 fold by ultrafiltration using Amicon Ultra MW 10.000 membranes (Millipore, Bedford, USA). UDP-sugar hydrolase activity was determined at 37 °C in a coupled spectrophotometric assay essentially as described before [53]. Briefly, reactions of the mixture containing 35 mM Tris–HCl, pH 8.0, 35 mM MgCl_2_, 3.1 μM glucose-1,6-bisphosphate, 0.7 mM NADP^+^, rabbit muscle phosphoglucomutase (1 U/ml) and *Leuconostoc mesenteroides* glucose-6-phosphate dehydrogenase (2.5 U/ml) were started by the addition of 1.4 mM UDP-glucose to the final volume of 1 ml. Glucose-1-phosphate formed by the reaction of UDP-sugar hydrolase was converted to glucose-6-phosphate and subsequently to 6-phosphogluconate by coupling of phosphoglucomutase and glucose-6-dehydrogenase, and the concomitant formation of NADPH (ε 340 nm = 6.3 mM^−1^ cm^−1^) was measured at 340 nm.

### Construction of transcriptional fusions and chloramphenicol acetyltransferase (CAT) assays

Different parts of the upstream region of the *pstSCAB* operon were amplified using the primers respectively named pstsR, pstsR0, pstsR1, pstsR2, pstsR3, pstsF0, pstsF1, pstsF2, pstsF3, pstsRc, pstsRm, pstsFc and pstsFm (Table 3) and cloned into the corynebacterial promoter-probe vector pET2 [54]. The vector pGEM-T (Table 1) was used for subcloning. The correct sequence of the cloned promoter fragments was verified by sequencing (AGOWA, Berlin, Germany). The constructed promoter-probe vectors were introduced into *C. glutamicum* WT as well as into the Δ*phoRS* mutant by electroporation using the following conditions: 25 μF, 600 Ω and 2.5 kV/cm (Bio-Rad Gene Pulser Xcell, Bio-Rad Laboratories, Hercules, Canada). After electroporation, 1 ml BHI/sorbitol medium was added immediately to the sample [55]. The cell suspension was exposed to 46 °C for 6 min and incubated at 30 °C for 90 min for regeneration. The CAT assays were performed as described previously [56].

### DNA affinity chromatography

The purification of DNA-binding proteins was performed essentially as described previously [57]. Briefly, *pstS* promoter fragments were generated by PCR using genomic DNA from *C. glutamicum* and the primer pair pstsR0/pstsF0bio. Primer pstsF0bio was tagged with biotin via a TEG linker (Operon, Cologne, Germany). Unincorporated oligonucleotides were removed by the Qiaquick PCR purification kit (Qiagen, Hilden, Germany). About 100 pmol of biotin-labeled PCR product was coupled to 5 mg of Dynabeads streptavidin (Dynal, Oslo, Norway) and free DNA was removed by magnetic separation. The coupled Dynabeads were stored at 4 °C. Cultures (900 ml) of *C. glutamicum* were grown on CGXII minimal medium, harvested at an optical density at 600 nm (OD600) of about 4, washed with 1 volume of TN buffer (50 mM NaCl, 50 mM Tris–HCl, pH 7.6) and suspended in 6 ml of TGED buffer (50 mM Tris–HCl (pH 7.6), 1 mM dithiothreitol, 10 mM MgCl_2_, 1 mM EDTA, 10 % (v/v) glycerol, 10 μM phenylmethylsulfonyl fluoride). The resuspended cell pellet was passed six times through a French pressure cell (SLM Amino, Spectronic Instruments, Rochester, NY) at 207 MPa. Cellular debris was removed by centrifugation at 8,000 g and 4 °C for 10 min and at 15,000 g and 4 °C for 60 min. Directly before incubation with the *C. glutamicum* crude extracts and the coupled Dynabeads, the beads were equilibrated with 300 μl of binding buffer (20 mM Tris–HCl pH 7,5, 1 mM EDTA, 10 % (v/v) glycerol, 0.01 % (v/v) Triton X-100, 100 mM NaCl and 1 mM dithiothreitol) for 2 min. The crude extract (about 6 ml) and 500 μg genomic DNA from *C. glutamicum* were incubated with the coupled Dynabeads for 1 h at room temperature with enough shaking to prevent sedimentation of the paramagnetic beads (150 rpm). Subsequently, the reaction was transferred into microcentrifuge tubes, washed once with 1 ml of TGED buffer, twice with 1 ml of TGED buffer including 400 μg of chromosomal DNA from *C. glutamicum* and finally with 1 ml of TGED buffer. Proteins bound to the immobilized DNA were eluted by washing the beads twice with 350 μl of elution buffer (TGED buffer containing 2 M NaCl). The eluates were pooled, concentrated and desalted with Microcon 3 microconcentrators (Millipore, Bedford, USA) and analysed by denaturing PAGE [51]. Gels were stained subsequently using a colloidal Coomassie blue staining kit (Novex, Frankfurt/Main, Germany).

### MALDI-TOF mass spectrometry

For peptide mass fingerprinting, the protein band of interest was cut out from gels and subjected to in-gel digestion with trypsin essentially as described previously [58]. Briefly, gel pieces were washed twice with 750 μl of 0.1 M ammonium bicarbonate in 30 % (v/v) acetonitrile for 10 min. The destained and shrunken gel pieces were vacuum-dried for 20 min in a conventional vacuum centrifuge and subsequently rehydrated with 6 μl of 3 mM Tris–HCl (pH 8.8) containing trypsin (10 ng/μl). After 20 min, 6 μl of 3 mM Tris–HCl (pH 8.8) without trypsin was added. Digestion was allowed to proceed overnight at room temperature. Peptides were then extracted by sequential addition of 6 μl of water and 10 μl of 0.1 % (v/v) trifluoroacetic acid in 30 % (v/v) acetonitrile. A total of 0.5 μl of the resulting peptide solution was mixed on a stainless steel sample plate with 0.5 μl of a saturated μ-cyano-4-hydroxy-trans cinnamic acid solution in 50 % (v/v) acetonitrile – 0.1 % (v/v) trifluoroacetic acid. Close external calibration using calibration mixtures 1 and 2 of a Sequazyme peptide mass standard kit (Applied Biosystems, Weiterstadt, Germany) was performed. Samples were analyzed manually in positive-reflector mode with 20 kV of accelerating voltage and 63 % grid voltage; the delay time was set at 125 ns. Data acquisition and analysis were performed using Voyager Control Panel software (version 5.0) and Voyager Data Explorer software (version 3.5) (Applied Biosystems). The generated mass lists and MS-Fit were used to search the National Center for Biotechnology Information (NCBI) database [59].

### Overproduction and purification of RamB

The RamB fusion protein was prepared essentially as described previously [41, 60]. Briefly, *E. coli* Bl21 (DE3) carrying the plasmid pET29*-ramB*-his was grown at 30 °C in 500 ml LB with 50 μg/ml kanamycin to an OD of 0.5 before adding 1 mM isopropyl ß-D-thiogalactoside. Four hours after induction, cells were harvested by centrifugation and stored at – 20 °C. For cell extract preparation, thawed cells were resuspended in 10 ml of TNGI5 buffer (20 mM Tris/HCl, pH 7.9, 300 mM NaCl, 5 % (v/v) glycerol, 5 mM imidazol) containing 1 mM diisopropylfluorophosphate and 1 mM phenylmethylsulfonyl fluoride. The cell suspension was passed six times through a French pressure cell (SLM Amino, Spectronic Instruments, Rochester, NY) at 207 MPa. Cell debris and intact cells were removed by centrifugation for 10 min at 5,000 g amd 4 °C, and the cell-free extract was subjected to centrifugation again for 1 h at 15,000 g and 4 °C. After centrifugation, the supernatant was purified by nickel affinity chromatography using Ni-NTA agarose (Novagen, San Diego, USA). The column was washed with TNGI20 and TNGI50 buffer (which contained 20 mM or 50 mM imidazol). The RamB protein was eluted with TNGI200 buffer (which contained 200 mM imidazol). Fractions containing RamB were pooled, and the elution buffer was exchanged against BS buffer (100 mM Tris/HCl, 20 % (v/v) glycerol, 100 mM KCl, 20 mM MgCl_2_, 1 mM EDTA, pH 7.5). From 250 ml of culture, ~ 4 mg of RamB was purified to apparent homogeneity (Fig. 3b).

### Gel mobility shift assays

Gel shift assays with RamB were prepared as described previously [60]. Briefly, overexpressed and purified RamB was mixed with the putative target promoter *pstS* (RF0) or promoter fragments (R0F0, R1F0, R2F0, R3F0, RF4, FcRc, FmRc, FcRm and FmRm) (124 bps – 507 bps, final concentrations 61 nM – 15 nM) (Figs. 4, 5) in a total volume of 20 μl. The binding buffer contained 100 mM Tris/HCl, 20 % (v/v) glycerol, 100 mM KCl, 20 mM MgCl_2_, 1 mM EDTA, pH 7.5. Approximately 40 nM of a nontarget promoter fragment (P_cg0527_, P_*dtxR*_ or R3F0) (Figs. 4, 5) were added as a negative control. After incubation for 30 min at room temperature, the samples were separated on a 10 % native polyacrylamide gel at room temperature and 170 V using 1x TBE (89 mM Tris base, 89 mM boric acid, 2 mM EDTA) as electrophoresis buffer. The gels were subsequently stained with Sybr Green I (Sigma, Rödermark, Germany) and photographed.

### DNA microarray analysis

Total RNA was isolated from exponentially growing cells by using the RNAeasy system (QIAGEN, Hilden, Germany) with on-column DNase I treatment prepared as described [61]. Quantity and quality of purified RNA was analyzed by UV-spectrometry and stored at −20 °C until use. DNA microarrays are based on PCR products of *C. glutamicum* genes [62]. Synthesis of fluorescently labelled cDNA from total RNA, microarray hybridization, washing and gene expression analysis were carried out as described previously [61–63]. The data are available as Gene Expression Omnibus GSE67012 data set at http://www.ncbi.nlm.nih.gov/geo/.
